# Maturation of the Female Pharyngeal Airway from Adolescence to Adulthood

**DOI:** 10.3390/jcm13020434

**Published:** 2024-01-12

**Authors:** Chun-Ming Chen, Han-Sheng Chen, Pei-Jung Chen, Kun-Jung Hsu

**Affiliations:** 1 School of Dentistry, College of Dental Medicine, Kaohsiung Medical University, Kaohsiung 80756, Taiwan; komschen@gmail.com (C.-M.C.); tabbyguy@gmail.com (H.-S.C.); 2 Department of Oral and Maxillofacial Surgery, Kaohsiung Medical University Hospital, Kaohsiung Medical University, Kaohsiung 807506, Taiwan; 3 Dental Department, Kaohsiung Municipal Siao-Gang Hospital, Kaohsiung 81253, Taiwan; 4 Department of Dentistry, Kaohsiung Medical University Hospital, Kaohsiung 80756, Taiwan

**Keywords:** female, pharyngeal airway space, adolescence, adulthood

## Abstract

Background: The present study aimed to investigate developmental changes in the female pharyngeal airway from adolescence to adulthood, considering variations in the anatomical structures related to the airway dimensions. Methods: Lateral cephalograms of 214 females were analyzed and categorized into five developmental stages: early adolescence (10–13 years), middle adolescence (14–17 years), late adolescence (18–21 years), early adulthood (22–30 years), and middle adulthood (31–50 years). The focus of the analysis included the point A-Nasion-point B (ANB) angle, tongue pharyngeal airway space (TPS), epiglottis pharyngeal airway space (EPS), soft palate airway space (SPS), and the horizontal and vertical positions of the hyoid bone. Results: The ANB angle exhibited significant variation across groups, being significantly larger in the early-adolescence group (4.22°) compared to the middle-adolescence, late-adolescence, and early-adulthood groups. The TPS and EPS were significantly shorter in the early-adolescence group. Negative correlations were observed between the ANB angle and the lengths of the pharyngeal airway spaces (SPS, TPS, and EPS). The horizontal and vertical positions of the hyoid bone remained stable after middle adolescence. Conclusion: The maturation of the ANB angle and pharyngeal airway in females seems nearly completed during middle adolescence (14–17 years). Additionally, a significant and negative correlation was identified between the ANB angle and the lengths of various pharyngeal airway spaces (SPS, TPS, and EPS). The horizontal and vertical positions of the hyoid bone showed stability after middle adolescence.

## 1. Introduction

The pharynx, a crucial anatomical structure situated in the neck, serves as a conical conduit connecting the oral and nasal cavities to the esophagus and trachea, playing a vital role in both swallowing and breathing processes [1,2]. Adolescence marks a period of significant physiological changes, encompassing alterations in height, weight, body structure, and circulatory and respiratory systems, largely driven by hormonal fluctuations [3,4]. Schendel et al. [5], utilizing cone-beam computed tomography, explored the growth and development of the pharyngeal airway in a diverse age group spanning from 6 to 60 years. Their findings delineated a pattern where the size and length of the pharyngeal airway undergo expansion until the age of 20, followed by a variable period of stability. Subsequently, a gradual reduction in the size of the pharyngeal airway ensues, accelerating notably after the age of 50. This suggests that the growth and development of the pharynx reach near completion during adolescence. Recker et al. [6] delved into the bone mass of healthy White women during early adulthood, identifying the peak bone mass around the age of 30. Following this stage, bone-building processes decelerate, with women being more susceptible to bone mass loss compared to men. Notably, individuals with respiratory diseases exhibited significantly lower bone mineral density than the general population [7].

In light of the dearth of reports on developmental changes in the pharyngeal airway in Asian females, the current study aims to bridge this gap by investigating the maturation of the female pharyngeal airway and associated anatomical structures from adolescence to adulthood. Given the relevance of the hyoid bone’s position to both tongue placement and pharyngeal airway size, we specifically examine its role in influencing these dimensions. Furthermore, we explore factors impacting pharyngeal airway size and their interrelationships.

## 2. Materials and Methods

A cephalometric unit (PM 2002-CC; Planmeca Inc., Helsinki, Finland) was employed to position patients in a natural head posture with centric occlusion. Lateral cephalograms were collected from 214 female patients at the dental department of Kaohsiung Medical University Hospital. The inclusion criteria were as follows: (1) healthy condition, (2) age ≥ 10 years, and (3) no history of orthodontic treatment. Patients with craniofacial symptoms or deformities, a history of craniofacial surgery, or facial trauma were excluded.

**Figure 1:**
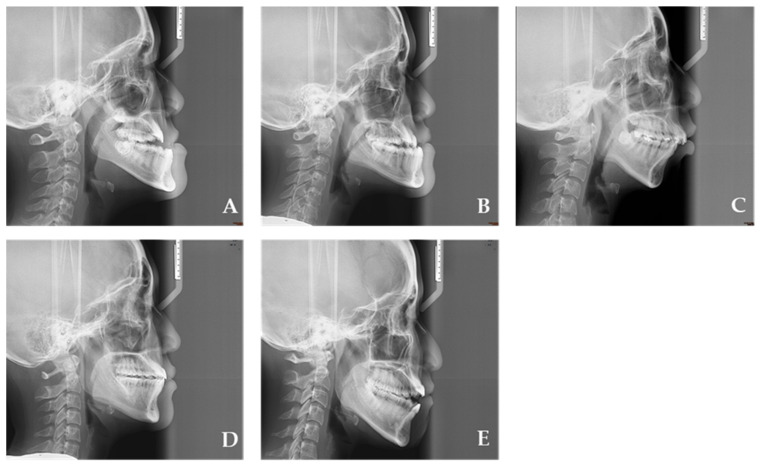
(**A**): Group 1 (early adolescence), (**B**): Group 2 (middle adolescence), (**C**): Group 3 (late adolescence), (**D**): Group 4 (early adulthood), and (**E**): Group 5 (middle adulthood).

The patients were categorized into five groups representing different developmental stages (Figure 1): Group 1 (early adolescence, 10–13 years), Group 2 (middle adolescence, 14–17 years), Group 3 (late adolescence, 18–21 years), Group 4 (early adulthood, 22–30 years), and Group 5 (middle adulthood, 31–50 years). Additionally, skeletal relationships were classified into three classes: Class I (ANB angle = 0° to 4°), Class II (ANB angle > 4°), and Class III (ANB angle < 0°).

**Figure 2:**
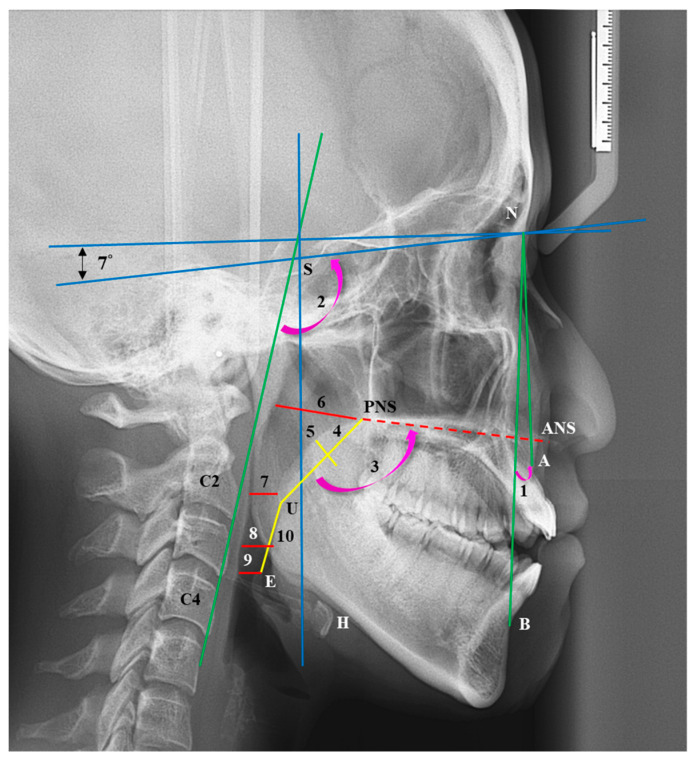
Cephalometric landmarks. S: sella; N: nasion; A point; B point; ANS: anterior nasal spine; PNS: posterior nasal spine; H: hyoid bone; C2: The most anterior inferior point of the second cervical vertebra; C4: The most anterior inferior point of the fourth cervical vertebra; U: uvula; E: epiglottis. Reference lines: The *X* axis in the X-rays was set as 7° upward of the line connecting the S and N, using N as the fulcrum. The line passing through S perpendicular to the *X* axis was considered to be the *Y* axis. Angular and linear measurements. 1: ANB angle; 2. C2C4-SN angle; 3: Palatal angle; 4: SPL (soft palate length); 5: SPW (soft palate width); 6: NPS (nasal pharyngeal airway space); 7: SPS (soft palate pharyngeal airway space); 8: TPS (Tongue pharyngeal airway space); 9: EPS (epiglottis pharyngeal airway space); 10: UE distance.

By using a 0.3 mm pencil, C.-M.C. manually identified cephalometric landmarks on tracing paper, as outlined in our previous study [8]. The cephalometric landmarks and angles are presented as follows (Figure 2):

S: Sella (the midpoint of the pituitary fossa);
N: Nasion (the most anterior point of the nasofrontal suture in the median plane);
A: The most concave point on the anterior edge of the maxilla;
B: The most concave point on the anterior edge of the mandibular symphysis;
ANS: Anterior nasal spine (the most anterior and superior spine of the maxilla);
PNS: Posterior nasal spine (the most posterior and superior spine of the maxilla);
H: The most anterior and superior point of the hyoid bone;
C2: The most anterior inferior point of the second cervical vertebra;
C4: The most anterior inferior point of the fourth cervical vertebra;
U: The lowest point of the uvula;
E: The uppermost point of the epiglottis;
ANB angle: The angle formed by point A, the nasion (N), and point B;
Palatal angle: The internal angle between the length of the soft palate and the ANS–PNS line;
C2C4-SN angle: The angle between the line passing through C2 to C4 and the SN line.

Reference lines were established with the *X*-axis set 7° upward of the line connecting S and N, and the *Y*-axis passing through S perpendicular to the *X*-axis.

The dimensions of the pharyngeal airway and soft palate are defined as follows:

Nasal pharyngeal airway space (NPS): Extending from ANS and PNS to the posterior wall of the pharynx;
Soft palate pharyngeal airway space (SPS): Shortest distance from the soft palate to the posterior pharyngeal wall;
Tongue pharyngeal airway space (TPS): Shortest distance from the tongue to the posterior wall of the pharynx;
Epiglottis pharyngeal airway space (EPS): Distance from the apex of the epiglottis to the posterior pharyngeal wall;
Soft palate length (SPL): Length of the soft palate;
Soft palate width (SPW): Width of the soft palate;
UE length: Distance between U and E.

Twenty radiographs were randomly selected and identified twice by C.-M.C. with a 1 week interval, and the reliability rates were assessed through intraclass correlation coefficients. All of the reliability rates were above 0.90. Data analysis was performed using SPSS Statistics version 20 (IBM; Chicago, IL, USA). Multiple intergroup comparisons were conducted using a one-way analysis of variance, and post hoc comparisons were done using the least significant difference. A Pearson correlation analysis was employed to determine the significance of intergroup differences in the pharyngeal airway space, head and neck position, and hyoid bone position. A *p* value of <0.05 indicated statistical significance. This study was approved by the human investigation review committee of Kaohsiung medical university hospital (KMUHIRB-E(II)-20180200).

## 3. Results

Table 1 presents the distribution of the 214 participants across the five groups based on developmental stages. Group 1 had 39 individuals (mean age: 10.97 ± 1.04 years), Group 2 had 27 individuals (mean age: 15.93 ± 0.87 years), Group 3 had 54 individuals (mean age: 19.80 ± 1.12 years), Group 4 had 66 individuals (mean age: 24.65 ± 2.14 years), and Group 5 had 28 individuals (mean age: 34.75 ± 4.45 years). The skeletal pattern classification included Class I with 102 participants (Group 1: 16; Group 2: 9; Group 3: 26; Group 4: 38; and Group 5: 13), Class II with 66 participants (Group 1: 20; Group 2: 10; Group 3: 14; Group 4: 14; and Group 5: 8), and Class III with 46 participants (Group 1: 3; Group 2: 8; Group 3: 14; Group 4: 14; and Group 5: 7).

In terms of skeletal patterns, Group 1 exhibited a significantly higher mean ANB angle (4.22°) compared to Groups 2, 3, and 4. Group 1 also had a significantly higher mean palatal angle (130.79°) than the other four groups. However, the C2C4-SN angle did not significantly differ among the groups.

Table 2 shows that the SPL was significantly shorter in Group 1 (32.28 mm) than in the other groups. The SPW did not significantly vary among the groups. The vertical position of the hyoid bone was significantly more superior in Group 1 compared to the other groups. However, the horizontal position of the hyoid bone did not significantly differ among the groups. Additionally, the UE length did not significantly differ among the groups.

Table 3 presents the mean lengths of various pharyngeal airway spaces across the five groups. The NPS length did not show a significant difference among the five groups. This suggests that the nasal pharyngeal airway space remains relatively consistent across early adolescence to middle adulthood in the studied sample. The SPS length was significantly shorter in Group 1 (early adolescence, 9.05 mm) compared to the other three groups (Group 2: 11.95 mm, Group 3: 11.32 mm, and Group 4: 10.45 mm). This indicates that the soft palate pharyngeal airway space undergoes growth and development, with a significant increase observed from early adolescence to middle adulthood. The TPS length was significantly shorter in Group 1 (early adolescence, 9.85 mm) compared to the other four groups. This suggests that the tongue pharyngeal airway space experiences growth and elongation as individuals progress from early adolescence to middle adulthood. The EPS length was significantly shorter in Group 1 (early adolescence, 5.91 mm) compared to the other four groups. Similar to the TPS, this indicates that the epiglottis pharyngeal airway space undergoes developmental changes, with a significant increase observed from early adolescence to middle adulthood.

In Table 4, the ANB angle displayed significant negative correlations with the SPS, TPS, and EPS lengths. The C4C2-SN angle showed significant positive correlations with the TPS and EPS lengths. The palatal angle had significant negative correlations with the SPS, TPS, and EPS lengths. The SPW exhibited a significant positive correlation with the NPS length and a significant negative correlation with the EPS length. The SPL had significant negative correlations with the lengths of the PS and UE. The horizontal position of the hyoid bone had a significant negative correlation with the UE length. Finally, the vertical position of the hyoid bone displayed significant positive correlations with the lengths of the TPS, EPS, and UE.

## 4. Discussion

Puberty, a biological process denoting the initiation of sexual maturation subsequent to early childhood, manifests with diverse initiation ages among individuals of the juvenile population. For most girls, puberty begins between the ages of 8 and 13 years, with peak growth occurring between the ages of 10 and 14 years. Adult height is reached between the ages of 15 and 16 years [9,10]. Adolescence is a developmental stage characterized by both physical and psychological maturity. Sexual maturity ratings (SMRs) [8], commonly known as Tanner stages [11], are widely employed to assess the physical development of adolescents through the established five stages of adolescence spanning prepuberty to adulthood. SMRs are used to evaluate the development of secondary sexual characteristics. Pubic hair and breasts are staged individually because their maturation rates vary. Tanner stage 5 represents the final stage of puberty, signifying the completion of the development and maturation of a woman’s body [11]. While many women may reach their average adult body size by age 16, there are some who may not reach their final adult body size until age 20 [9,10]. Notably, growth and development in Asian women tend to occur 1–2 years later than those in White women. Based on clinical observations, we determine adolescence as a period between 10 and 21 years, while adulthood is defined as 22 years onwards.

Hunter [12] investigated the relationship between facial growth, height, and skeletal maturity during adolescence, and found that in most people, the peak of facial growth coincides with the peak of height growth, and that mandibular development correlates with height. Our findings were consistent with those of Hunter [12]. During early adolescence, individuals showed an ANB angle of 4.22° in this study. Maxillary development is still ongoing at this point, and the mandibular bone is not yet fully developed. Early adolescence seems to have a larger ANB angle than the subsequent four developmental stages. The ANB angle was found to be 1.52° during middle adolescence, which is when the mandibular bone development is most active. The development of the mandible is completed in early adulthood. The ANB angle is generally constant during middle adolescence through middle adulthood. It is noteworthy that the ANB angle did not differ significantly between the stages of middle adolescence, late adolescence, early adulthood, and middle adulthood.

The C2C4-SN angle showed no significant changes among the five groups. However, although not significant, it gradually increased from 104.85° in early adolescence to 108.48° in middle adulthood. As individuals progress through different stages of life, they undergo several changes in their physical attributes. As an individual matures, they tend to experience growth in their height and overall size. Consequently, there is also an increase in their physical activity. These developments result in an escalated demand for adequate airway space within their respiratory system. By adjusting the head position upward, the C2C4-SN angle increases to meet the demands of increased activity, better respiratory capacity, and maintaining airway patency. Hsu et al. [8] investigated the changes in the pharyngeal airway space in primary school students. In that study, 93 elementary school students were divided into three age groups (Group 1: 7–8 years old, Group 2: 9–10 years old, and Group 3: 11–12 years old). The C2C4-SN angle gradually increased from 98.5° in Group 1 to 102.1° in Group 2 and to 105.4° in Group 3. As a result, the C2C4-SN angle in Group 3 was significantly greater than that in Group 1. Clinical observations are consistent with this finding. Most children have their head and neck slightly tilted forward, while adolescents are more upright. Although the SPW didn’t change much over the five stages, the SPL was significantly shorter in early adolescence than in any of the subsequent developmental stages. It is possible that the growth of the soft palate is why the palatal angle was significantly smaller in middle adolescence than in early adolescence. Due to gravity, the uvula is slightly pulled forward, resulting in a decrease in the palatal angle. In particular, the SPL and the palatal angle were not significantly different at the four stages from middle adolescence to middle adulthood, suggesting that the development of the hard and soft palates is almost complete by middle adolescence.

The hyoid bone is horseshoe-shaped and located in the neck between the mandible and thyroid cartilage [13,14]. According to Werner et al. [15], the hyoid bone starts off in developing infants at the level of the second and third vertebral junctions. It then gradually descends until the upper end of the hyoid is at the level of the third and fourth vertebral junctions by the age of 2 years. The hyoid bone continues to descend slowly throughout childhood and into early adulthood. The hyoid and associated muscles play a significant role in maintaining the size of the pharyngeal airway by varying their position with the mandible. Across the five developmental stages, the horizontal position of the hyoid bone did not differ significantly. The hyoid bone moved forward but not significantly, leading to a gradual increase in horizontal distance. Similarly, in accordance with Werner et al. [15], the vertical position of the hyoid bone was considerably higher in early adolescence than in the other four developmental stages. The hyoid bone undergoes a significant downward shift starting from middle adolescence in response to craniofacial development. By middle adolescence, the development of the hyoid bone is complete. As a result, the vertical position of the hyoid bone remained constant throughout the four developmental stages from middle adolescence through middle adulthood. A longitudinal study by Jeans et al. [16] showed that the size of the nasopharyngeal airway shows an acceleration of growth from the age of 9 onwards. This growth peaked around age 13 and continued slowly until age 19. Our observations were consistent with this trend, as the length of the NPS increased slowly and showed no significant differences across the five developmental stages. Myslik et al. [17] reported that the average length of the SPS was 6.52–9.23 mm at 6–9 years of age, 8.70–8.91 mm at early adolescence, and 8.85–9.9 mm at mid-adolescence. Our study found that early adolescence had an SPS length of approximately 9.05 mm, which was similar to that of Mislik et al. [17]. Furthermore, the length of the SPS during middle adolescence was approximately 11.95 mm, which was longer than that reported by Mislik et al. [17].

Taylor et al. [18] conducted a longitudinal cephalogram study in 32 individuals (16 male and 16 female) aged 6–18 years. Two periods of accelerated change (6–9 years and 12–15 years) and two periods of quiescence (9–12 years and 15–18 years) were identified. Hsu et al. [8] reported that the TPS length was significantly longer in children aged 11–12 years (10.2 mm) than in those aged 7–8 years (7.8 mm). In our study, the TPS of the early adolescent group was significantly shorter than the other four groups. The TPS length did not significantly differ across the four developmental stages from middle adolescence through middle adulthood. The lack of significant differences in TPS length across the four developmental stages from middle adolescence through middle adulthood, is attributed to the maturation of the mandible and tongue, indicating a stage (middle adolescence) of near completion in growth. In summary, our results are consistent with those of Taylor et al. [18].

From birth until 3 years old, the larynx experiences rapid growth, but then slows down during puberty. As early adolescents progress, the laryngeal structure gradually descends to around the fourth or fifth segment of the cervical spine [19]. In the study, early adolescence had a significantly smaller EPS than the other four groups. However, the EPS did not significantly differ across those other four groups. By middle adolescence, EPS growth is almost complete due to the maturation of the larynx. Furthermore, we found a significant positive relationship between age and EPS length.

It was found that there were significant negative correlations between the ANB angle and the lengths of the SPS, TPS, and EPS. This mention of a larger airway space being associated with a smaller ANB angle has potential clinical implications, especially in orthodontics and airway assessments. Furthermore, there are significant positive correlations between the C2C4-SN angle and the lengths of TPS and EPS, suggesting that the influence of the C2C4-SN angle on the TPS and EPS lengths was more noticeable compared to its effects on the lengths of NPS and SPS.

The SPS, TPS, and EPS lengths had significant negative correlations with the palatal angle, implying a larger airway space when the angle is smaller. This effect is similar to that of the ANB angle. Furthermore, we found that a larger angle of the ANB indicated a larger angle of the palatal angle. Despite the weak correlation, a significant positive correlation was observed among the palatal angle and NPS length. Additionally, the SPL had a negative correlation with the lengths of the SPS and UE. There is no observed correlation between the horizontal position of the hyoid bone and the length of the pharyngeal airway. In contrast, a positive correlation is noted between the vertical position of the hyoid bone and the lengths of the TPS and EPS. It can be inferred that the vertical position of the hyoid bone has a more pronounced effect on specific aspects of the pharyngeal airway, such as the TPS and EPS, compared to its horizontal position.

This paper presents valuable insights into how the female pharyngeal airway changes and progresses throughout adolescence and adulthood. However, certain limitations of the study should be acknowledged. The sample distribution of the developmental groups was not evenly distributed. The analysis did not include a late adulthood group (age > 50). Class II malocclusion is frequently characterized by a larger ANB angle, which may have caused bias in the results. To accurately determine changes in the ANB angle throughout childhood and adulthood, a sample must be screened for orthodontic issues such as malocclusions to avoid selection bias. Because of the variety of females, the dimensions of the pharyngeal airway space can vary between ethnic groups.

## 5. Conclusions

According to the research findings, female individuals’ development of pharyngeal airway spaces reaches a stage of near maturation by middle adolescence (14–17 years). The ANB angle showed the most significant but negative correlation with changes in pharyngeal airway dimensions. This means that the smaller the ANB angle, the larger the pharyngeal airway space. These findings have important implications for both orthodontic treatment planning and a broader understanding of pharyngeal airway development in women.

## Figures and Tables

**Table 1:** Female characteristics from adolescence to adulthood in the one-way analysis of variance.

	Age		ANB Angle		C2C4-SN Angle		Palatal Angle	
	Mean	SD	Mean	SD	Mean	SD	Mean	SD
Group 1 (*n* = 39)	10.97	1.04	4.22	3.03	104.85	8.36	130.79	4.79
Group 2 (*n* = 27)	15.93	0.87	1.52	4.60	106.31	7.63	123.91	6.31
Group 3 (*n* = 54)	19.80	1.12	2.03	4.35	107.74	7.56	125.95	7.87
Group 4 (*n* = 66)	24.65	2.14	2.25	3.56	106.51	7.20	126.02	5.71
Group 5 (*n* = 28)	34.75	4.45	2.73	4.35	108.48	7.17	126.05	6.03
*p* value	<0.001		0.040		0.288		<0.001	
Multiple comparisons	5 > 4> 3 > 2 > 1		1 > 2, 1 > 3, 1 > 4		NS		1 > 2, 1 > 3, 1 > 4, 1 > 5	

*n*: number of participants; Group 1: early adolescence; Group 2: middle adolescence; Group 3: late adolescence; Group 4: early adulthood; Group 5: middle adulthood; Multiple comparisons: Statistically significant, *p* < 0.05; NS: not significant.

**Table 2:** Dimension and location of the soft palate and the hyoid bone from adolescence to adulthood in the one-way analysis of variance.

	SPW		SPL		Hyoid H		Hyoid V		UE	
	Mean	SD	Mean	SD	Mean	SD	Mean	SD	Mean	SD
Group 1 (*n* = 39)	8.14	1.62	32.28	3.80	9.86	7.40	104.87	8.87	21.49	5.06
Group 2 (*n* = 27)	8.48	1.91	34.61	4.30	13.23	6.60	114.79	6.87	23.26	4.66
Group 3 (*n* = 54)	8.44	1.56	34.24	4.19	12.69	7.43	115.16	6.35	23.06	5.30
Group 4 (*n* = 66)	8.36	1.62	35.13	4.03	12.96	8.40	114.71	6.86	23.58	5.57
Group 5 (*n* = 28)	8.25	1.52	35.09	3.71	13.57	9.10	113.84	5.91	22.45	5.13
*p* value	0.899		0.009		0.256		<0.001		0.370	
Multiple comparisons	NS		5 > 1, 4 > 1, 3 > 1, 2 > 1		NS		5 > 1, 4 > 1, 3 > 1, 2 > 1		NS	

*n*: number of participants; Group 1: early adolescence; Group 2: middle adolescence; Group 3: late adolescence; Group 4: early adulthood; Group 5: middle adulthood; SPW: soft palate width; SPL: soft palate length; Hyoid H (Horizontal); Hyoid V (Vertical); UE: distance between uvula and epiglottis; Multiple comparisons: Statistically significant, *p* < 0.05; NS: not significant.

**Table 3:** Pharyngeal airways from adolescence to adulthood in the one-way analysis of variance.

	NPS		SPS		TPS		EPS	
	Mean	SD	Mean	SD	Mean	SD	Mean	SD
Group 1 (*n* = 39)	23.43	4.57	9.05	3.16	9.85	3.02	5.91	2.50
Group 2 (*n* = 27)	24.82	3.05	11.95	4.30	12.65	4.28	8.06	3.61
Group 3 (*n* = 54)	24.80	3.31	11.32	2.98	12.50	3.07	8.10	2.48
Group 4 (*n* = 66)	24.76	3.32	10.45	2.84	11.58	2.88	7.87	2.45
Group 5 (*n* = 28)	23.92	2.89	10.23	3.11	12.08	3.12	8.20	2.88
*p* value	0.257		0.002		0.001		0.001	
Multiple comparisons	NS		2 > 1, 3 > 1, 4 > 1		5 > 1, 4 > 1, 3 > 1, 2 > 1		5 > 1, 4 > 1, 3 > 1, 2 > 1	
			2 > 4, 2 > 5					

*n*: number of participants; Group 1: early adolescence; Group 2: middle adolescence; Group 3: late adolescence; Group 4: early adulthood; Group 5: middle adulthood; NPS: nasal pharyngeal airway space; SPS: soft palate pharyngeal airway space; TPS: tongue pharyngeal airway space; EPS: epiglottis pharyngeal airway space; Multiple comparisons: Statistically significant, *p* < 0.05; NS: not significant.

**Table 4:** Pearson correlation (r) test for related angles and linear distances in all females.

	Age	ANB	C4C2-SN	Palatal	SPW	SPL	Hyoid H	Hyoid V
		Angle	Angle	Angle				
NPS	0.046	0.072	0.067	0.346 *	0.185 *	0.105	−0.110	0.069
SPS	0.019	−0.331 *	−0.029	−0.183 *	0.083	−0.338 *	0.037	0.096
TPS	0.097	−0.324 *	0.172 *	−0.212 *	−0.081	−0.084	0.036	0.148 *
EPS	0.208 *	−0.305 *	0.165 *	−0.208 *	−0.148 *	0.045	0.047	0.188 *
UE	0.081	0.190 *	0.092	0.106	0.113	−0.180 *	−0.231 *	0.315 *

SPW: soft palate width; SPL: soft palate length; Hyoid H (Horizontal); Hyoid V (Vertical); NPS: nasal pharyngeal airway space; SPS: soft palate pharyngeal airway space; TPS: tongue pharyngeal airway space; EPS: epiglottis pharyngeal airway space; UE: distance between uvula and epiglottis; *: Statistically significant, *p* < 0.05.

## Data Availability

The data used to support the findings of this study are available from the corresponding author upon request.
